# Circulating Plasmablasts from Chronically Human Immunodeficiency Virus-Infected Individuals Predominantly Produce Polyreactive/Autoreactive Antibodies

**DOI:** 10.3389/fimmu.2017.01691

**Published:** 2017-12-06

**Authors:** Hongyan Liao, Yangsheng Yu, Song Li, Yinshi Yue, Chuanmin Tao, Kaihong Su, Zhixin Zhang

**Affiliations:** ^1^Department of Laboratory Medicine, West China Hospital, Sichuan University, Chengdu, China; ^2^Department of Pathology and Microbiology, University of Nebraska Medical Center, Omaha, NE, United States; ^3^Qilu Hospital of Shandong University, Jinan, China; ^4^Internal Medicine, University of Nebraska Medical Center, Omaha, NE, United States; ^5^Eppley Research Institute, University of Nebraska Medical Center, Omaha, NE, United States; ^6^Department of Pediatrics, West China Second University Hospital, State Key Laboratory of Biotherapy, Ministry of Education Key Laboratory of Birth Defects, Sichuan University, Chengdu, China

**Keywords:** human immunodeficiency virus, B cell, plasmablast, polyreactive antibody, autoreactive antibody, Ig heavy, repertoire, VH replacement

## Abstract

Understanding the B-cell response during chronic human immunodeficiency virus (HIV) infection is essential for eliciting broad and potent neutralizing antibodies (Abs). In this study, we analyzed the plasmablast repertoire of chronically HIV-infected individuals in combination with antiretroviral therapy (ART). Among the obtained 72 recombinant monoclonal antibodies (mAbs), 27.8% weakly bound to HIV gp140 and were non-neutralizing. Remarkably, 56.9% were polyreactive and 55.6% were autoreactive. The prominent feature of being polyreactive/autoreactive is not limited to anti-gp140 Abs. Furthermore, these polyreactive/autoreactive Abs displayed striking cross-reactivity with DWEYS in the *N*-methyl-d-aspartate receptor (NMDAR), and this binding induced SH-SY5Y cell apoptosis. We also found higher frequencies of VH4-34 utilization and VH replacement in the plasmablast repertoire of chronically HIV-infected individuals, which may contribute to the generation of poly/autoreactive Abs. Taken together, these data demonstrate that circulating plasmablasts in chronically HIV-infected individuals experienced with ART predominantly produce poly/autoreactive Abs with minimal anti-HIV neutralizing capacity and potential cross-reactivity with autoantigens. This may represent another dysfunction of B cells during chronic HIV infection.

## Introduction

Human immunodeficiency virus (HIV) infection affects millions of people worldwide (1). Designing effective vaccines to induce broad and potent neutralizing antibodies (bnAbs) remains the ultimate approach for preventing HIV transmission (2). HIV-1 directly infects and depletes CD4^+^ T cells (3–6), which destroys T follicular helper cell function and impairs the T-dependent B-cell response (7–10). Although B cells are not the direct targets of HIV replication, previous evidence has revealed that multiple B-cell dysfunctions directly or indirectly result from HIV replication (11, 12). In the initial stage, HIV infection induces polyclonal activation and depletion of follicular B cells (13–15). Later, chronic HIV infection alters B-cell subsets through loss of memory B cells (16), exhaustion of B cells (17), and clonal expansion of plasmablasts (15, 18). B cells are the main components of the humoral response and responsible to produce neutralizing antibodies (Abs). A better understanding of the B-cell response is therefore essential for inducing bnAbs to control HIV infection.

Early studies of a panel of well-characterized bnAbs showed that many of them are polyreactive or autoreactive against common self-antigens (19). This may partially explain the rarity of such bnAbs, as they may have been eliminated during early B-cell development due to their cross-reactivity with self-antigens. Recent analyses of gp140-binding memory B cells in HIV-infected patients with bnAbs showed that up to 75% of gp140-reactive Abs are poly/autoreactive (20). Based on this observation, it was proposed that bnAbs might be selected from a preexisting pool of polyreactive B cells and that polyreactivity may increase the binding affinity to HIV antigens through hetero-ligation (20).

Plasmablasts are B cells that can differentiate into antibody-secreting cells (ASCs) while still proliferating (12). The absolute count and relative frequency of plasmablasts in the circulation directly reflect the body’s active immune response (21–23). Previous work has demonstrated a high proportion of plasmablasts, accounting for upwards of 50% of all circulating B cells, in early HIV infection; although the frequency is diminished relative to the early phase, it is still elevated compared with that of healthy donors during the chronic phase (22). However, the Ab profiles dictated by the altered frequency of peripheral circulating plasmablasts remain unclear. Accordingly, the current study was undertaken to investigate the functional Ab repertoire of circulating plasmablasts in chronically HIV-infected individuals in combination with antiretroviral therapy (ART). We found that circulating plasmablasts in HIV-infected individuals experienced with ART predominantly produce polyreactive/autoreactive Abs regardless of their anti-HIV neutralizing capacity. This is likely to represent a previously unrealized dysfunction of B cells during chronic HIV infection. Additionally, we demonstrated that such non-neutralizing polyreactive Abs cross-reacted with a key autoantigen target, the DWEYS consensus peptide present in the *N*-methyl-d-aspartate receptor (NMDAR), which is widely expressed on neuronal cell membranes. Such cross-reactivity induces neuroblastoma SH-SY5Y cell apoptosis *in vitro*. We also revealed a bias favoring the utilization of the autoimmune-associated VH4-34 and VH1-2 genes and VH replacement. These data have key implications for our knowledge of plasmablast responses in chronically HIV-infected individuals with ART experience and the role of non-neutralizing polyreactive Abs in HIV/AIDS pathogenesis.

## Materials and Methods

### Human Specimens

Peripheral blood samples from chronically HIV-infected individuals were obtained from the Specialty Care Center at the University of Nebraska Medical Center (UNMC), Omaha, NE, USA. All study subjects were free from other ongoing infections and were chronically infected (>2 years) with stable viral load and CD4^+^ T-cell count after ART. The controls were all healthy people excluded from ongoing infections and chronic diseases, and were matched with the HIV-infected individuals by age, gender, and race. CD4^+^ T-cell counts and frequency of circulating plasmablasts in CD19^+^ B cells in all study subjects were analyzed. A summary of the demographics of all study subjects is shown in Table S1 in Supplementary Material.

### Ethics Statement

All human samples were collected with written informed consent under protocols approved by the UNMC Institutional Review Board (IRB).

### Cell Preparation and Sorting

Mononuclear cells (PBMC) were purified by Ficoll gradient centrifugation from 20 mL of peripheral blood samples. PBMCs were stained with FITC-conjugated anti-human CD3 Abs, BV421-conjugated anti-human CD19 Abs, BV510-conjugated anti-human CD20 Abs, PE-conjugated anti-human CD27 Abs, and APC-conjugated anti-human CD38 Abs (BD Biosciences, San Jose, CA, USA). After staining, HIV-positive samples were fixed with 4% formaldehyde (Sigma-Aldrich, St. Louis, MO, USA) for 30 min at room temperature (RT) to eliminate live virus. Samples were analyzed at the UNMC FACS core facility. Circulating plasmablasts were identified as CD3^−^CD19^low^CD20^low^CD27^hi^CD38^hi^ cells by FACS Aria (BD Biosciences) and sorted into 96-well PCR plates at one cell/well with 10 µL of lysis buffer (10 mM Tris–HCl pH8.0, 10 U RNasin (Promega, Madison, WI, USA). The sorted single cells were directly subjected to RT-PCR amplification or stored at −80°C for future analysis. Gating and analysis of frequencies of plasmablasts were performed using by BD FACSDIVA™ software (Figure S1 in Supplementary Material).

### Amplification of Ig Genes by Single-Cell PCR and Expression of Recombinant Monoclonal Antibodies (mAbs)

Single-cell PCR analysis was applied in the case of seven healthy controls and eight HIV-infected individuals (Table S2 in Supplementary Material). The 96-well PCR plate containing sorted cells was incubated at 65°C for 4 h to reverse the crosslinking. RT-PCR was performed using the QIAGEN OneStep RT-PCR kit following the manufacturer’s instructions. Two rounds of PCR reactions were used to amplify IgH, Igκ, or Igλ genes from single cells as previously described (24). To facilitate subcloning, a third round of PCR was performed. Third-round PCR products with unique restriction enzyme digestion sites were subcloned into corresponding Igγ1, Igκ, or Igλ expression vectors. The resulting plasmids were sequenced, and paired IgH and IgL genes from the same well were cotransfected into 293T cells [American Type Culture Collection (ATCC) CRL-3216] to express recombinant mAbs. Culture supernatant was collected 7 days after transfection and Abs were purified using Protein A Agarose beads (ThermoFisher Scientific, Madison, WI, USA).

### Sequence Analysis

The Ig heavy (IgH) and light (IgL) variable (V) region sequences were analyzed using the ImMunoGeneTics information system (IMGT)/V-Quest program (http://www.imgt.org/IMGT_vquest/share/textes/) (25) to assign VH, DH, and JH genes. B cells were considered to belong to the same clone on the basis of identical V, D, and J gene segment usage and CDR3 length for both heavy- and light-chain Ig genes. Unique sequences (GenBank accession MG385193–MG385264) were analyzed, whereas identical sequences were eliminated. Potential VH replacement products were identified using VH replacement analyzer-1 (VHRFA1) (26).

### Assays for Ab Reactivity

The polyreactivity, anti-gp140 reactivity, and DWEYS reactivity of the obtained recombinant mAbs were analyzed by ELISA. ELISA plates were coated with double-stranded DNA (dsDNA) and single-stranded DNA (ssDNA) in PBS, lipopolysaccharide (LPS), insulin (Sigma-Aldrich, St. Louis, MO, USA), DWEYS in coating buffer (pH = 7.6) at 10 µg/mL, and YU2 gp140 and gp140 trimer (HIV-1 clade B) (Immune Tech, Suzhou, Jiangsu, China) in PBS at 1 µg/mL at 4°C overnight. Coated plates were blocked with 100 µL PBST/BSA buffer (150 mM NaCl, 50 mM Tris–HCl, 1 mM EDTA, 2.0% BSA, and 0.05% Tween-20) for 2 h at RT, followed by incubation with 100 µL of recombinant mAbs (1:4 serially diluted in PBS with 2% BSA) at RT for 1 h. After washing four times with PBST, biotin-conjugated goat anti-human IgG Fc Abs (Jackson ImmunoResearch Laboratories Inc., West Grove, PA, USA) were added (100 µL, 1:4,000 dilution) to each well and incubated at RT for 1 h. Bound Abs were detected with horseradish peroxidase (HRP)-conjugated streptavidin (Jackson ImmunoResearch Laboratories Inc., West Grove, PA, USA, 1:8,000 dilution). For color development, 3,3′,5,5′-tetramethylbenzidine (TMB, Kirkegaard & Perry Laboratories, Gaithersburg, MD, USA) was added and incubated for 10 min. The reaction was stopped with 0.5 M H_2_SO_4_, and the absorbance at 450 nm (OD450) was measured on a POLARstar Omega plate reader (BMG LABTECH, Cary, NC, USA). The PG9, PG16, 2F5, and 4E10 Abs (gift from Dr. Yongjun Guan) were used as standards for gp140 reactivity and polyreactivity testing. For anti-gp140 reactivity, the cut-off value was set as 3 SEM above the average reactivity of control Abs. For reactivity against ds-DNA, ss-DNA, insulin, LPS, and DWEYS, the cut-off value was set as 3 SEM above the average reactivity of control Abs.

Indirect immunofluorescent ANA assay (IIFA) was performed using the HEp-2 ANA kit (MBL International, Woburn, MA, USA) according to the manufacturer’s instructions. Briefly, HEp-2 cell-coated slides were incubated with purified Ab (25 µg/mL) for 30 min at RT. After washing with PBS for 4 times, slides were incubated with FITC-labeled anti-human IgG Abs (Kirkegaard & Perry Laboratories, Gaithersburg, MD, USA) for 30 min at RT. Slides were washed with PBS and visualized under an Olympus 1 × 81 inverted fluorescence microscope (Olympus, Tokyo, Japan). Images were captured with the same exposure setting and analyzed using the Slidebook5 program (Intelligent-Imaging). Specific staining patterns, such as nuclear and cytoplasmic, were documented.

### Neutralization Assay

Neutralization of a selected panel of representative virus isolates by mAbs was measured using pseudovirus infection of TZM-bl cells as previously described (27).

The following reagents were obtained from the NIH AIDS Reagent Program: the TZM-bl cell line; the plasmid pSG3^ΔEnv^ to produce HIV pseudoviruses; and plasmids containing HIV envelopes of SF162, BaL.26, and WITO4160.33. Briefly, 5 µg of plasmids carrying the functional Env clone from selected virus isolates and 10 µg of the backbone pSG3^ΔEnv^ recombinant plasmids were transfected into HEK 293T/17 cells (5 × 10^6^ cells/mL). Virus supernatants were harvested after 48 h of incubation at 37°C with 5% CO_2_. p24 protein in each virus sample was quantified by using the AlphaLISA HIV p24 Biotin-Free detection kit (Perkin Elmer, Waltham, MA, USA), and input virus was normalized to 5–10 ng/mL for the following TZM-bl cell assay. Pseudotyped viruses containing the Env of amphotropic murine leukemia virus (aMLV) were used as the negative control. Then, 10 µL of mAbs fivefold serially diluted from a starting concentration of 50 µg/mL were incubated with 40 µL of replication-competent virus samples in duplicate for 30 min at 37°C in 96-well clear flat-bottom black culture plates (Greiner Bio-One, Monroe, NC, USA). TZM-bl cells were added at a concentration of 10,000 cells per 20 µL to each well in DMEM containing 75 µg/mL DEAE-dextran and 1 µM indinavir. Cell-only and virus-only controls were included on each plate. Plates were incubated for 24 h at 37°C in a 5% CO_2_ incubator, after which the volume of culture medium was adjusted to 200 µL by adding complete DMEM containing indinavir. 48 h post-infection, 100 µL was removed from each well and 100 µL of SpectraMax Glo Steady-Luc reporter assay (Molecular Devices, LLC., CA, USA) reagent was added to the cells. To determine luciferase activity, cells were lysed using M-PER Mammalian Protein Extraction Reagent (Pierce, Rockford, IL, USA) and approximately 20 µL of each lysed sample was transferred to a microplate. Reporter Lysis Buffer (Promega, Madison, WI, USA) was added to each sample in the microplate and the light intensity was measured using a SpectraMax i3x multi-mode detection platform following the manufacturers’ instructions. Non-infected cells in wells were used to determine background luciferase activity, which was subtracted from the activity measured for all other samples. Relative light units (RLU) per milliliter were calculated by dividing the luciferase values by their corresponding dilutions. The amount of infectious virus was determined as RLU per nanogram of p24. The 50% inhibitory concentrations IC50 was defined as the Ab dilution that caused a 50% reduction in neutralization. IC50s of known bnAbs P9, PG16, 2F5, and 4E10 were also determined against the cross-clade panel of pseudoviruses (Table S3 in Supplementary Material).

### Immunofluorescence Staining and Flow Cytometry

SH-SY5Y cells (ATCC 2266) were cultured in DMEM medium with 10% heat-inactivated fetal bovine serum (FBS), 2 mM l-glutamine, 100 U/mL penicillin, 100 µg/mL streptomycin, and 25 mM HEPES. For immunofluorescence microscopy analyses, cells were subcultured in glass coverslips pre-coated with l-lysine. HIV201B2 and U78Ab3 were labeled with Dylight 488 (ThermoFisher Scientific, Bremen, Germany) according to the manufacturer’s instructions. For flow cytometry analyses, cells were fixed with 4% paraformaldehyde and stained with Dylight 488-labeled mAb (10 µg/mL) at RT, and recorded using an Olympus 1 × 81 inverted fluorescence microscope (Olympus, Tokyo, Japan). For SH-SY5Y cell competition-binding assessment, cells were coincubated with Dylight 488-labeled HIV201B2 and DWEYS with and increasing molar ratio, and recorded using a BD Accuri C6 system (BD Biosciences, San Jose, CA, USA).

### MTT Assay

SH-SY5Y cells cultured in DMEM medium were treated with HIV201B2 and U78Ab3 with increasing concentrations (2, 10, 50, and 250 µg/mL) for 24 h. Cell viability was measured using the MTT formazan (Sigma Aldrich, St. Louis, MO, USA) assays following the manufacturer’s instructions and reading the absorbance at 570 nm using a standard spectrophotometer.

### Statistical Analysis

Statistical analyses were performed using GraphPad Prism 5.0 (GraphPad Software, Inc., San Diego, CA, USA). For Ig gene repertoire, Ab reactivity, and amino acid (aa) usage, statistical analyses were performed using Fisher’s exact test. For the CD4^+^ T-cell count, the percentage of plasmablasts in CD19^+^ B cells, and the frequency of VH replacement, the statistical analyses were performed using the Mann–Whitney test. For IgH CDR3 length and VH mutation rate, the statistical analyses were performed using Student’s *t* test on condition that the *F*-test for equal variance was executed first and two-sided *P* > 0.05. The correlations of CD4^+^ T-cell count, viral load and plasmablast frequency, anti-dsDNA reactivity, and reactivity against other antigens were determined by Spearman’s ranked correlation test. Two-sided *P* ≤ 0.05 was considered statistically significant, and *P* < 0.0001 was considered extremely statistically significant.

## Results

### Reduced CD4^+^ T-Cell Counts and Elevated Plasmablast Proportion in Individuals with Chronic HIV Infection

We examined the CD4^+^ T-cell count and percentage of plasmablast in each study subject by flow cytometry. The CD4^+^ T-cell counts were significantly reduced in chronically HIV-infected individuals compared with those in healthy controls (Figure S2A in Supplementary Material; *P* < 0.0001 by Mann–Whitney test). The percentage of plasmablasts among CD19^+^ B cells (mean ± SD, 2.20 ± 0.18%) was significantly higher than that in healthy controls (mean ± SD, 0.47 ± 0.09%) (Figure S2B in Supplementary Material; *P* < 0.0001 by Mann–Whitney test). These results indicate ongoing immune responses to HIV infection. Plasmablast frequency did not correlate with CD4^+^ T-cell count (Figure S2C in Supplementary Material; *r* = 0.2964, *P* = 0.2834 by Spearman’s ranked correlation test) or viral load (Figure S2D in Supplementary Material; *r* = −0.1574, *P* = 0.5753 by Spearman’s ranked correlation test).

### Circulating Plasmablasts in Chronically HIV-Infected Individuals Producing Non-Neutralizing Low-Affinity Anti-gp140 Abs

**Figure 1:**
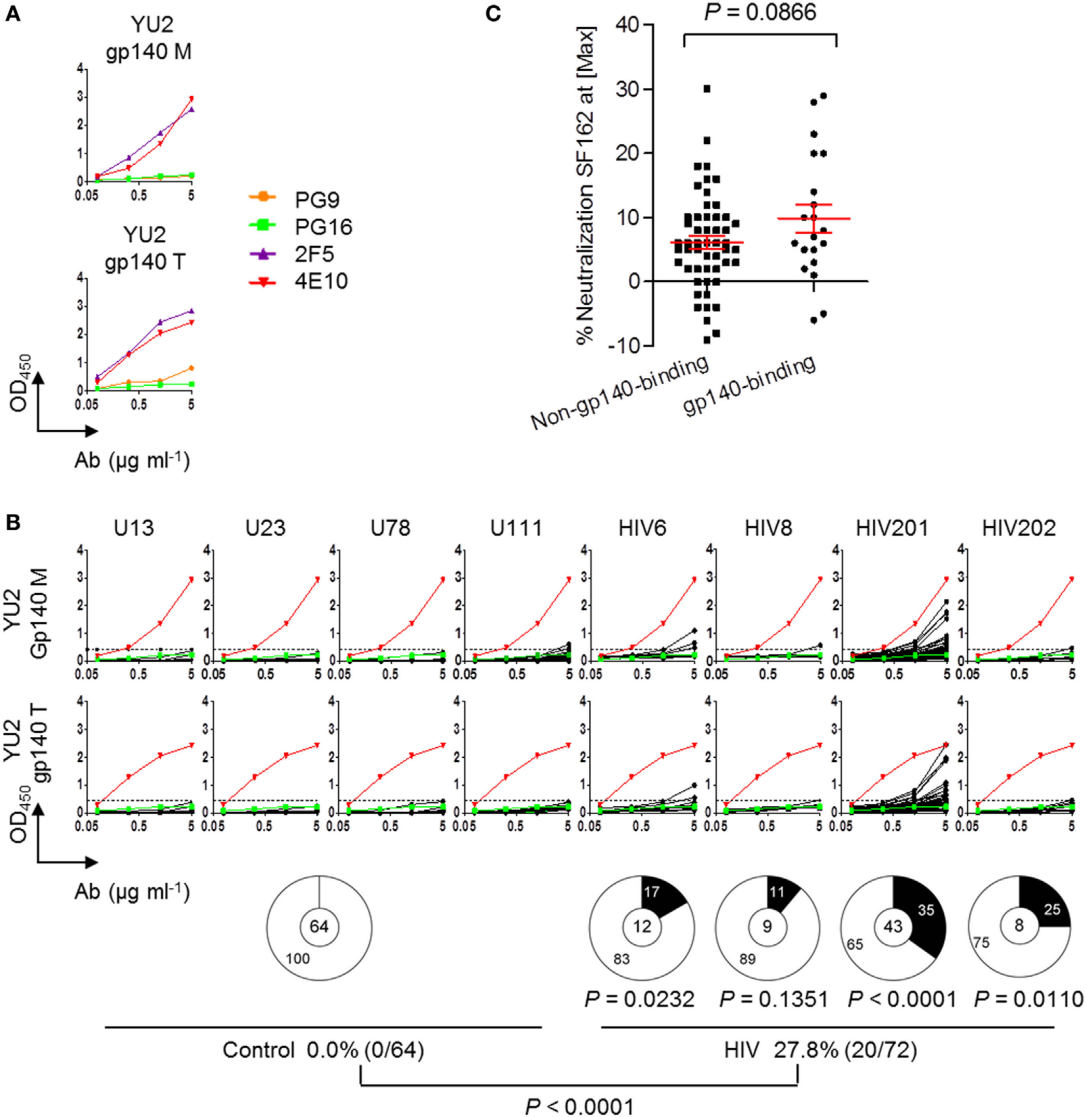
Circulating plasmablasts in chronically HIV-infected individuals producing low-affinity anti-gp140 antibodies (Abs). **(A)** ELISA results of known bnAbs (PG9, PG16, 4E10, and 2F5) against YU2 gp140 monomer and trimer. **(B)** ELISA results of the recombinant monoclonal antibodies (mAbs) derived from the plasmablasts of control and HIV-infected individuals binding to YU2 gp140 monomer and trimer. The dashed line represents the cut-off OD450_nm_ value for positive reactivity by the anti-gp140 test, which is 3 SEM above the average reactivity of control Abs. Each line with four dots represent individual Ab tested upon four serial dilutions. Green lines represent PG16 as the negative control. Red lines represent 4E10 as the positive control. Pie graphs show percentages of gp140-binding Abs in the control Ab pool and in each HIV-infected subject. Digits in pie centers represent total numbers of mAbs cloned from each individual. Digits in pie outer regions represent percentages of mAbs that are reactive with gp140 (black) and not reactive with gp140 (white). The bottom panel summarizes total frequencies of anti-gp140 Abs in four healthy controls and four HIV-infected individuals. *P*-value was determined by Mann–Whitney test. **(C)** Comparison of percentages of neutralized virus (SF162) at max concentration of Ab (50 μg/mL) applied in TZM-bl assay between non-anti-gp140 and anti-gp140 Abs. *P*-value was determined by unpaired *t*-test.

We expressed 64 mAbs and 72 mAbs from the circulating plasmablasts of four healthy controls and four chronically HIV-infected individuals, respectively (Table S2 in Supplementary Material). To determine the antigenic specificity of each mAb, we performed ELISAs against both HIV gp140 monomer and trimer (HIV-1 clade B, YU2). Well-known bnAbs 2F5, 4E10, PG9, and PG16 (28–30) were analyzed as standards to optimize the assay. PG9 and PG16 are the type of Ab that do not bind to monomeric gp120 but are capable of neutralizing a large proportion of viruses at low titers (30). Consistent with previous findings (30, 31), 2F5 and 4E10 were reactive with both gp140 monomer and trimer; PG9 was weakly reactive with gp140 trimer, whereas PG16 was not reactive with gp140 in either form (Figure 1A). Therefore, PG16 and 4E10 were used as the negative and positive control, respectively, in gp140-ELISA. The four HIV-infected individuals exhibited varying frequencies of Abs reactive against gp140, with a total of 27.8% of the anti-gp140 Abs (Figure 1B). This frequency was significantly higher than that in the control group (Figure 1B). However, the gp140-affinities of these Abs were extremely low. We included two of the most sensitive tier-1 clade B virus isolates (SF162 and BaL.26), and one moderately sensitive tier-2 clade B virus isolate (WITO4160.33) (32–34) (Table S3 in Supplementary Material). We tested the mAbs for neutralization *via* generation of pseudoviruses followed by TZM-bl cell infection assay. While none of them was capable of neutralize SF162, the gp140-binding and non-gp140-binding mAbs displayed no significant difference in neutralization potency (Figure 1C; *P* = 0.0866 by unpaired *t*-test). No Ab was identified to be able to neutralize other viruses tested (data not shown). Therefore, none of these mAbs seemed to be the PG9 and PG16 type, which bind to trimeric gp120 but not monomeric gp120, and have broad and potent neutralization activity. Taken together, these results suggest that mAbs cloned from randomly sorted plasmablasts of chronic HIV-infected individuals with ART experience were weak gp140-binding and non-neutralizing Abs.

### Circulating Plasmablasts in Chronically HIV-Infected Individuals Predominantly Producing Polyreactive Abs

**Figure 2:**
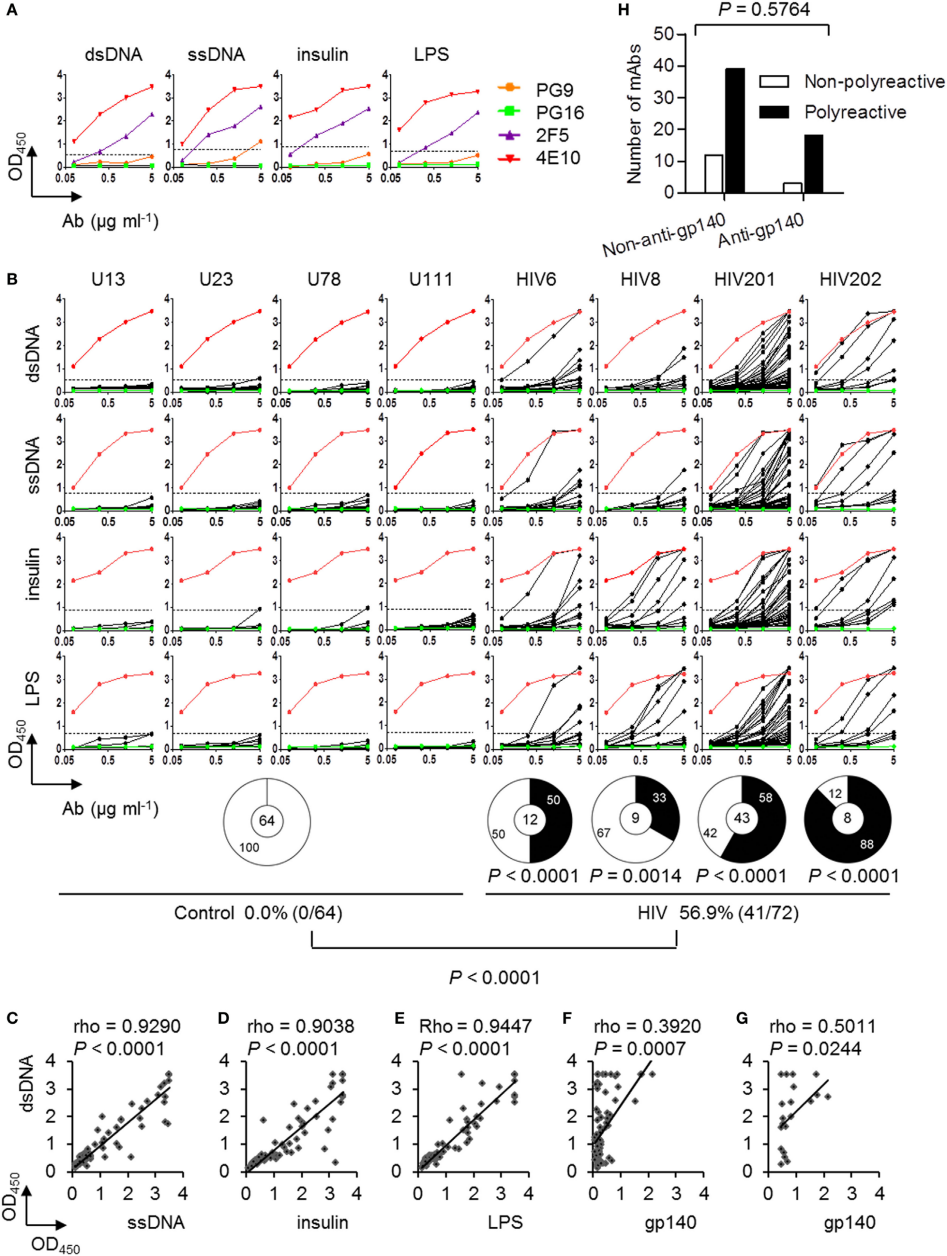
Polyreactivity of monoclonal antibodies (mAbs) cloned from circulating plasmablasts of control and chronically HIV-infected individuals. **(A)** ELISA results of known bnAbs (PG9, PG16, 4E10, and 2F5) against double-stranded DNA (dsDNA), single-stranded DNA (ssDNA), insulin, and lipopolysaccharide (LPS). **(B)** ELISA results of each recombinant mAbs derived from plasmablasts of control and HIV-infected individuals against dsDNA, ssDNA, insulin, and LPS. Red lines represent 4E10, as the positive control. Green lines represent PG16, as the negative control. Dashed lines represent the cut-off OD450_nm_ value for positive reactivity, which is 3 SEM above the average reactivity of control Abs. Pie charts summarize the frequency of polyreactive (black) and non-polyreactive (white) Abs. The percentages of polyreactive Abs in control and HIV-positive group are summarized in the bottom panel. *P*-values are in comparison with pooled Abs derived from the control donors. *P*-value was determined by Fisher’s exact test. **(C–G)** Correlations of the anti-dsDNA reactivities of the 72 recombinant mAbs from HIV-infected individuals with their reactivities against ssDNA **(C)**, insulin **(D)**, LPS **(E)**, and gp140 **(F)**. **(G)** Correlations of the anti-dsDNA reactivities with gp140-binding Abs only. rho and *P*-values were determined by Spearman’s ranked correlation test. **(H)** Numbers of non-polyreactive Abs among non-anti-gp140 and anti-gp140 Abs compared with polyreactive Abs. *P*-value was determined by chi-squared test.

Previous analyses of mAbs derived from HIV gp140-specific memory B cells showed that the majority (75%) were poly/autoreactive (20). To determine polyreactivity, we analyzed the plasmablast-derived mAbs from our study by ELISA using the same panel of antigens, i.e., dsDNA, ssDNA, insulin, and LPS. Abs reacting against at least two of these antigens were considered to be polyreactive (20). Previously identified bnAbs 2F5, 4E10, PG9, and PG16 (28–30) were analyzed as standards to optimize the assay. The bnAbs 2F5 and 4E10 were polyreactive, but PG9 and PG16 were not (Figure 2A). Notably, all mAbs in the present study were expressed from the accurately gated single plasmablasts of HIV-positive individuals without any preselection of antigenic specificity. Although the frequencies of polyreactive Abs varied among the four HIV-infected individuals (from 33 to 88%), they were all significantly elevated compared with those in controls (Figure 2B). We found that 56.9% of mAbs (41/72) from the HIV group were polyreactive (Figure 2B). Further analyses showed that the anti-dsDNA reactivity of the Abs derived from the HIV-infected individuals were strongly correlated with their reactivities against ssDNA (Figure 2C), insulin (Figure 2D), and LPS (Figure 2E) but were only weakly correlated with their reactivity against HIV gp140 (Figure 2F). When we focused on the gp140-binding Abs, their anti-dsDNA reactivity showed a moderate correlation with their gp140 reactivity (Figure 2G). Moreover, there was no significant difference in the percentages of polyreactive Abs between anti-gp140 and non-anti-gp140 mAbs (Figure 2H). Taken together, these results indicate that circulating plasmablasts in chronically HIV-infected individuals with ART experience predominantly produce polyreactive Abs regardless of their gp140 reactivity; among these polyreactive Abs, some of them bind weakly to gp140.

**Figure 3:**
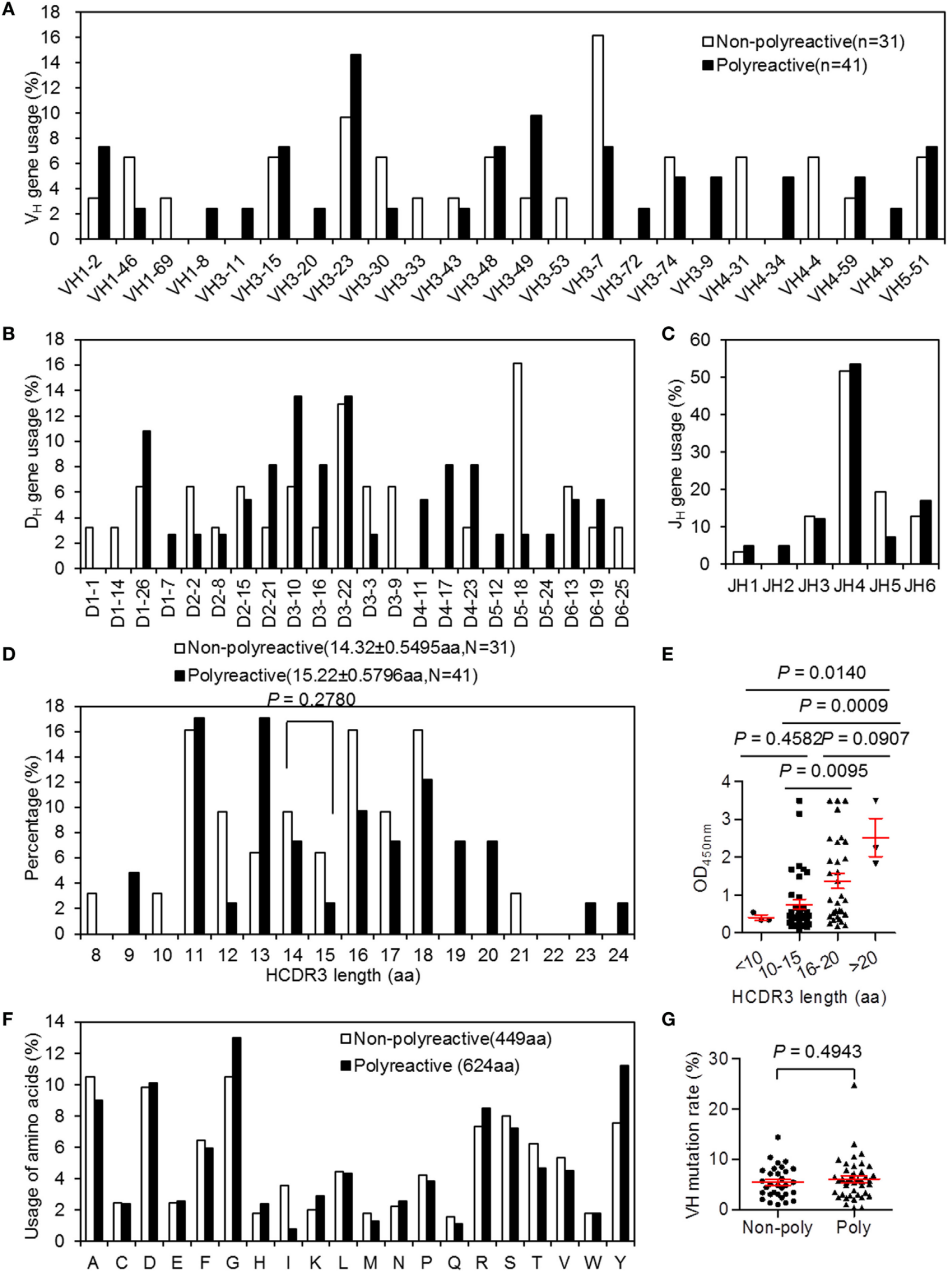
VH, DH, and JH gene usages, HCDR3 length and amino-acid usages, and VH mutation rates in Ig heavy (IgH) sequences of 72 monoclonal antibodies (mAbs) derived chronically HIV-infected individuals grouped by polyreactivity. **(A–C)** VH **(A)**, DH **(B)**, and JH **(C)** gene. **(D)** Distribution of IgH CDR3 lengths (aa, amino acid) of non-polyreactive Ab group and polyreactive Ab group. **(E)** Comparisons of OD values of dsDNA-ELISA within groups of different HCDR3 lengths. **(F)** Proportion of aa usages in HCDR3 regions and **(G)** rate of VH mutations between non-polyreactive Ab group and polyreactive Ab group. Red error bars indicate mean with SEM.

To identify molecular differences that might contribute to polyreactivity, we compared the 72 IgH genes expressed as mAbs from chronic HIV-infected individuals (Table S4 in Supplementary Material). The distributions of individual VH, DH, and JH genes of polyreactive and non-polyreactive Abs were examined. Unexpectedly, there were no preferential VH, DH, or JH gene segments (Figures 3A–C). The HCDR3 region is the canonical antigen-binding site with the most variability. Previous studies have shown that long and charged HCDR3 loops are associated with polyreactivity (35). We examined the variation in the HCDR3 length and aa content. For polyreactive Ab genes, the average HCDR3 is composed of ~15 aa and is slightly longer than the HCDR3 of genes encoding non-polyreactive Abs (Figure 3D; *P* = 0.2768 by unpaired *t*-test). To examine the contribution of HCDR3 length in more detail, we compared the OD values reflecting the strength of reactivity against dsDNA in groups of Abs derived from chronic HIV-infected individuals according to their HCDR3 length. Notably, Abs with longer HCDR3 were more reactive to dsDNA. A declining trend of OD values was seen as the HCDR3 length decreased (Figure 3E). The relative proportional distribution of aa was similar in the Ab repertoire of both groups (Figure 3F). To gain further insight into the mechanisms that might contribute to polyreactivity, we also analyzed the VH mutation rate. The non-polyreactive Abs and polyreactive Abs showed a similar rate of mutation in VH regions (Figure 3G). Overall, HCDR3 length is a prominent contributor to the polyreactivity characteristic of mAbs from chronic HIV-infected individuals.

### Circulating Plasmablasts in Chronically HIV-Infected Individuals Predominantly Producing Autoreactive Abs

**Figure 4:**
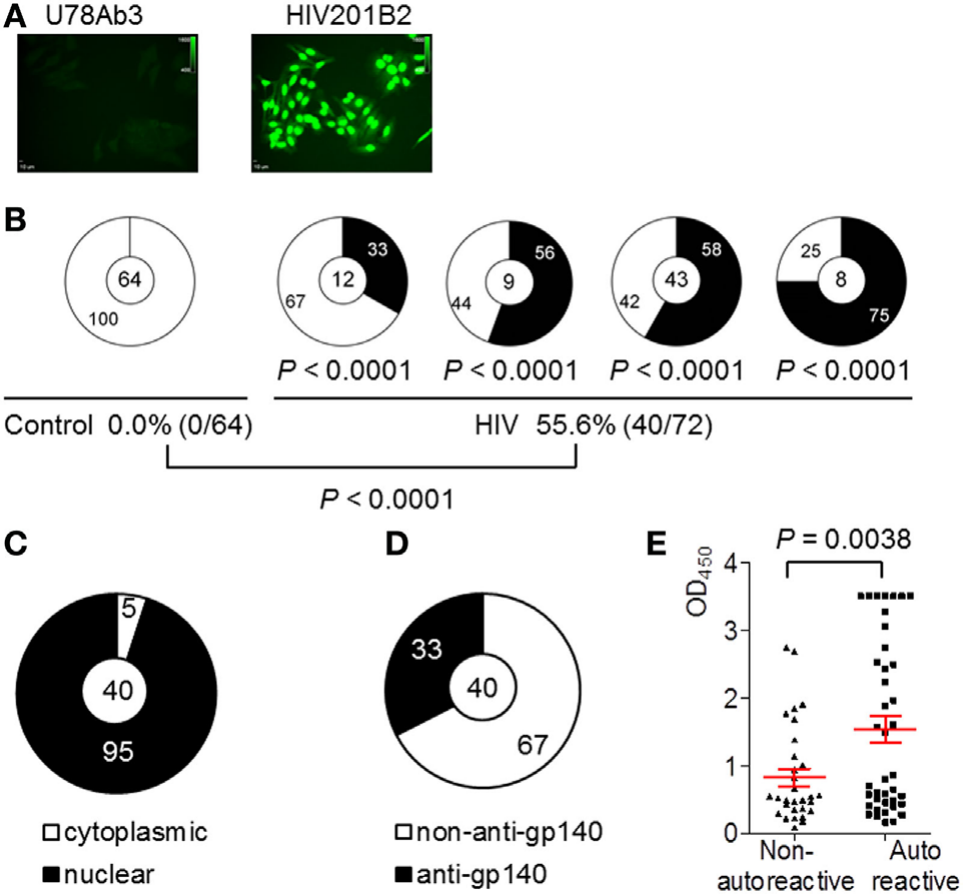
Autoreactivity of monoclonal antibodies (mAbs) cloned from circulating plasmablasts of control and chronically HIV-infected individuals. **(A)** Representatives of autoantibodies (ANA) patterns of non-autoreactive Ab U78Ab3 from control and autoreactive Ab HIV201B2 from patient (scale bar: 10 µm). **(B)** Shown are pie charts summarizing the frequencies of autoreactive (black) and non-autoreactive (white) Abs. The percentages of ANAs in control and HIV-positive group are summarized in the bottom panel. **(C)** Shown is pie graph summarizing the percentages of nucleus- (black) and cytoplasmic-reactive (white) Abs in total of 40 ANAs.**(D)** Shown is pie graph summarizing the percentage of gp140-binding Abs (black) and non-gp140-binding Abs (white) in total of 40 ANAs. **(E)** Comparison of OD450_nm_ values in dsDNA-ELISA between ANAs and non-ANA. Red error bars indicate mean with SEM.

Next, we performed HEp-2 cell-based IIFA to determine whether the recombinant mAbs derived from the plasmablasts of HIV-infected individuals were autoantibodies (ANAs). Among the 72 recombinant mAbs derived from the HIV-infected individuals, 40 (55.6%) reacted with fixed HEp-2 cells. This frequency was significantly higher than that in the control donors (Figure 4B). Notably, among these 40 ANAs, 95% (38/40) showed a homogeneous nuclear staining pattern, whereas only 5% (2/40) exhibited cytoplasmic antigen staining (Figures 4A,C), which reflected their dsDNA-binding abilities. Moreover, 32.5% (13/40) showed positive gp140-binding results (Figure 4D). Additionally, these 40 ANAs exhibited significantly stronger dsDNA-binding affinities than the 32 non-autoreactive ANAs (Figure 4E; *P* = 0.0038 by unpaired *t*-test). Thus, the mAbs derived from the circulating plasmablasts of chronically HIV-infected individuals prominently exhibit poly/autoreactive and non-neutralizing characteristics.

### Plasmablast-Derived Poly/Autoreactive mAbs from Chronically HIV-Infected Individuals Cross-Reacting with NMDAR and Inducing SH-SY5Y Cell Apoptosis

**Figure 5:**
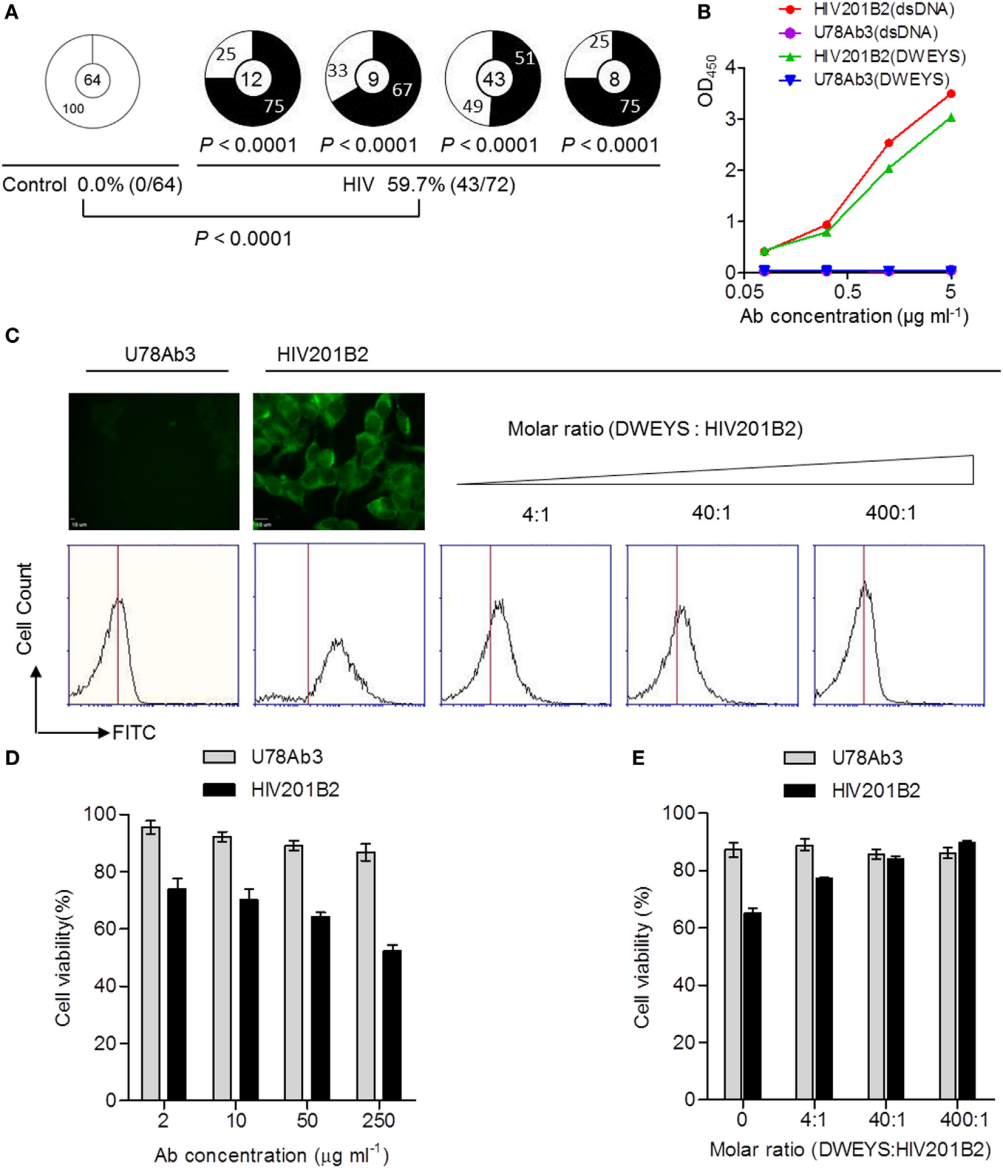
NMDAR-cross-reactivity and effect on SH-SY5Y cell viability of monoclonal antibodies (mAbs) cloned from circulating plasmablasts of chronically HIV-infected individuals. **(A)** Shown are pie charts summarizing the frequencies of DWEYS-cross-reactive Abs (black) and Abs without DWEYS-cross-reactivity (white). *P*-values are in comparison with pooled Abs derived from the control donors. The percentages of DWEYS-cross-reactive Abs in control and HIV-positive group are summarized at the bottom. **(B)** DWEYS- and dsDNA-ELISA-binding curves of two mAb representatives: U78Ab3, double negative, and HIV201B2, double positive. **(C)** The top-left panel shows the staining of HIV201P5B2 on the membrane of SH-SY5Y cells (scale bar: 10 µm). Histograms show the competitive binding of DWEYS to HIV201B2. Blank bars show **(D)** the decreasing cell viability as the concentration of HIV201B2 applied to treat SH-SY5Y cells goes up and **(E)** the increasing cell viability as the molar ratio of DWEYS to HIV201B2 applied to treat SH-SY5Y cells goes up. Gray bars show U78Ab3 as the control. Error bars indicate mean with SD.

The cross-reactivity of anti-dsDNA ANAs with the DWEYS peptide sequence present in NMDAR subunits is a recognized molecular mechanism of neuropsychiatric lupus (NPSLE) (36–38). To determine whether the poly/autoreactive mAbs share this cross-reactivity with NMDAR, we measured their DWEYS reactivity by ELISA. We found significantly higher frequencies of DWEYS-reactive mAbs in the four chronically HIV-infected individuals than in the controls (Figure 5A). We used HIV201B2 as a model NMDAR-reactive ANA and U78Ab3 as an isotype-matched control Ab. HIV201B2 showed high affinity to both dsDNA and DWEYS (Figure 5B) and was autoreactive with a homogeneous nuclear staining pattern (Figure 4A). We first determined by immunofluorescence staining that HIV201B2 bound robustly to SH-SY5Y cells, with staining on the outer membrane where NMDARs are supposedly expressed, which was not observed with U78Ab3 (Figure 5C, top left). We simultaneously recorded staining *via* flow cytometry; of note, as increasing amounts of DWEYS were applied, HIV201B2 showed a gradual loss of binding to SH-SY5Y cells (Figure 5C, bottom). This result provides solid evidence that HIV201B2 bound to SH-SY5Y cells *via* DWEYS. We continued to explore how this binding would affect the cells. Remarkably, HIV201B2 induced evident cell death as its concentration in the culture medium increased, whereas U78Ab3-treated cells exhibited a more normal cell viability after 24 h (Figure 5D). When the DWEYS peptide with increasing concentrations was applied together with the cross-reactive Ab, the cell toxicity was gradually blocked (Figure 5E). Several other mAbs were also tested, and similar effects were seen (Figure S3 in Supplementary Material). We therefore suspect that non-neutralizing anti-HIV mAbs with strong polyreactivity and autoreactivity, such as HIV201B2, may be pathological due to their cross-reactivity with autoantigens.

### Comparison of the IgH Repertoires of Plasmablasts of Chronically HIV-Infected Individuals and Controls

**Figure 6:**
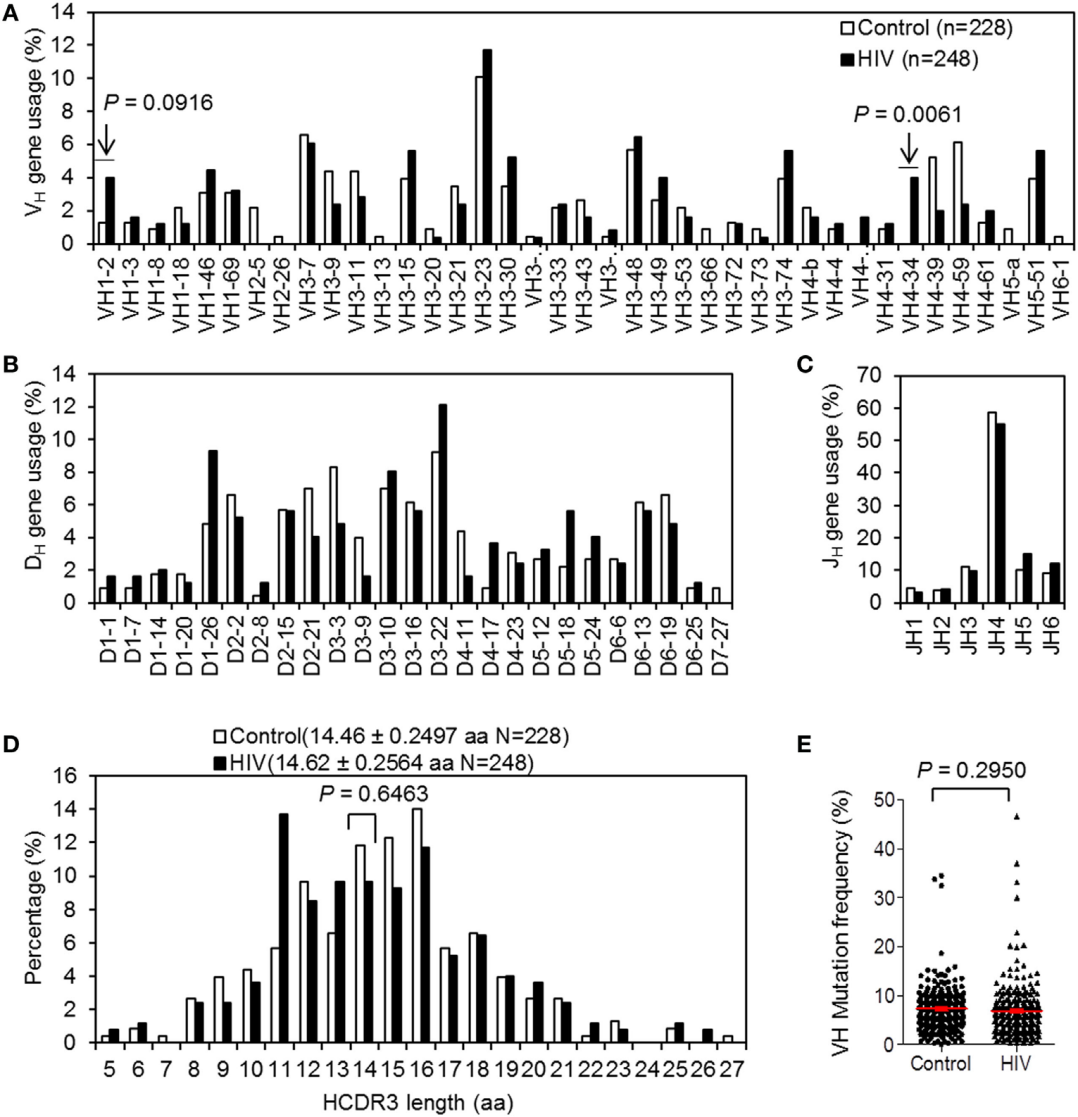
VH, DH, and JH gene usages, HCDR3 length, and VH mutation rate of Ig heavy (IgH) genes derived from plasmablasts controls and chronically HIV-infected individuals. **(A–C)** VH **(A)**, DH **(B)**, and JH **(C)** gene usages of the IgH genes. **(D)** Analyses of the IgH CDR3 lengths (amino acid, aa) of the predicted encoding Abs. **(E)** Comparison of VH somatic mutation rates between IgH genes derived from control donors and chronically HIV-infected individuals. Red error bars indicate mean with SEM. *P*-value was determined by unpaired *t*-test.

We obtained 248 unique IgH gene sequences from eight chronically HIV-infected individuals and 228 unique IgH gene sequences from seven control donors (Table S2 in Supplementary Material). Analyses of the potential VH, DH, and JH germline gene usages among IgH genes derived from both groups showed diversified repertoires (Figures 6A–C). The VH3-23 gene, the most often utilized VH gene in naïve and memory B-cell repertoires (39), was also the most frequently used in the plasmablast repertoires of both groups (Figure 6A). Notably, the frequency of VH4-34 utilization in IgH genes derived from HIV-infected individuals was significantly elevated (Figure 6A). VH1-2 gene utilization was marginally more frequent in the HIV group (Figure 6A). The heavy-chain CDR3 (HCDR3) is the antigen-binding region with the highest variability and often plays a decisive role in antigen binding. We did not find a significant difference in HCDR3 region length between the two groups (Figure 6D). Similarly, there was no significant difference in the VH mutation rate between repertoires of control and HIV-infected individuals (Figure 6E).

### Enrichment of VH Replacement Products in IgH Genes Derived from Plasmablasts of HIV-Infected Individuals

**Figure 7:**
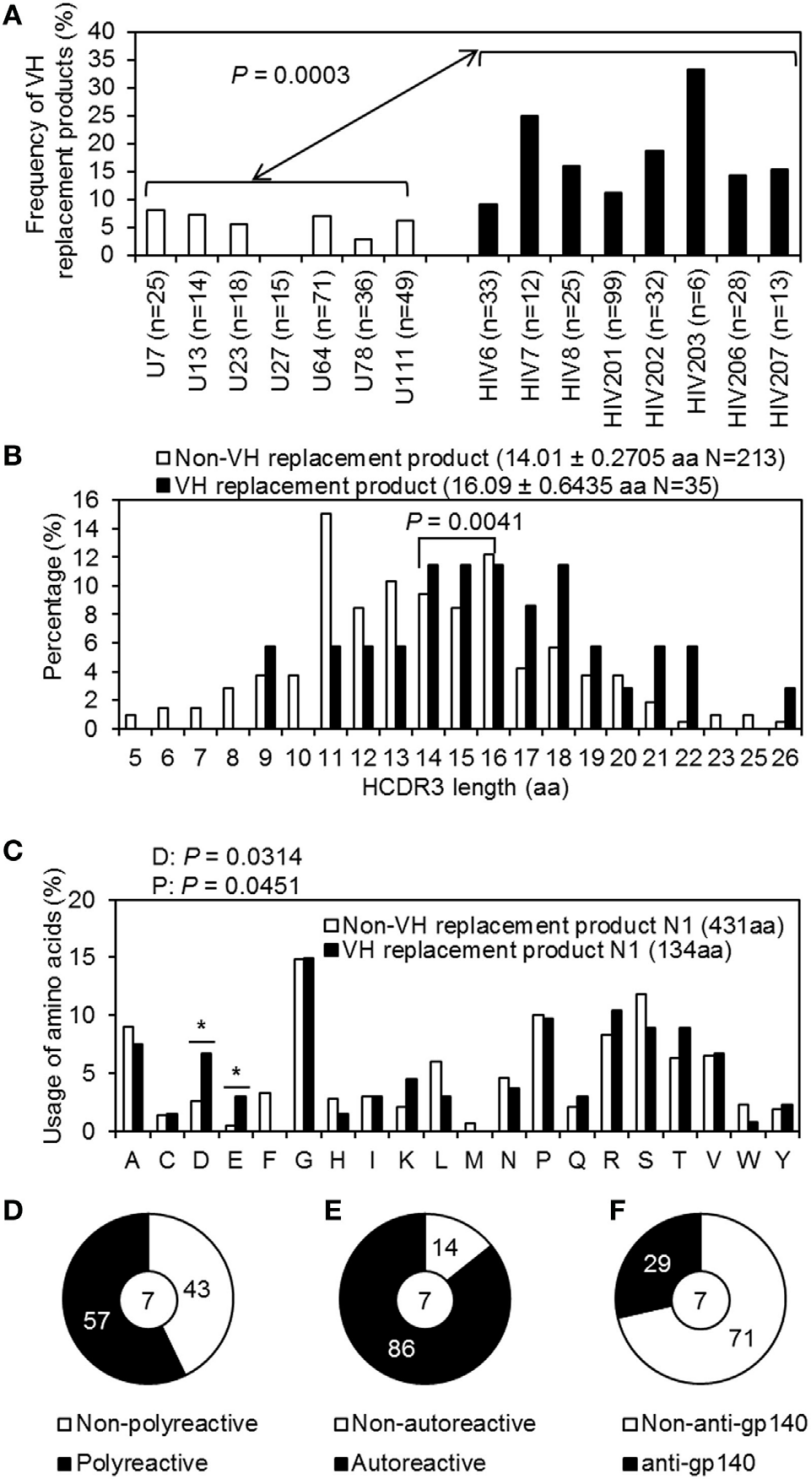
VH replacement analyses of Ig heavy (IgH) genes derived from plasmablasts of control and chronically HIV-infected individuals. **(A)** Shown is bar graph summarizing the frequencies of VH replacement products in IgH gene sequences derived from plasmablasts of each control donor (white bars) and HIV-infected individual (black bars). **(B)** Comparison of the IgH CDR3 lengths of non-VH replacement products (white bar; average ± SD: 14.01 ± 0.2705 aa) and VH replacement products (black bar; average ± SD: 16.05 ± 0.6439 aa). **(C)** Bar graph shows the frequency of aa contributed by the identified VH replacement footprints versus that in the N1 regions of non-VH replacement products. **(D–F)** Pie graphs show the percentage of polyreactive **(D)** autoreactive **(E)** and anti-gp140 **(F)** Abs encoded by VH replacement products.

Previous evidence has demonstrated that VH replacement contributes to the neutralization of B-cell autoreactivity and diversification of the Ab repertoire (40–42). VH replacement utilizes evolutionarily conserved cryptic recombination signal sequences (cRSSs) to generate novel VH rearrangements, with a short stretch of nucleotides retained as a VH “replacement footprint” at the VH–DH junction (N1) (43). We used the VHRFA program (26) to identify VH replacement footprints in the N1 regions (Table S5). In IgH genes derived from the plasmablasts of chronically HIV-infected individuals, VH replacement products were significantly enriched compared with the frequency in the control group (Figure 7A). These identified VH replacement products exhibited significantly longer CDR3 regions than the non-VH replacement products (Figure 7B) and preferentially contributed charged aa, such as Glu (D) and Asp (E), to the CDR3 regions (Figure 7C).

Among the 35 VH replacement products derived from HIV-infected individuals, seven were expressed as recombinant mAbs. Interestingly, 57.1% (4/7) of the Abs encoded by VH replacement products were polyreactive (Figure 7D) and 85.7% (6/7) were autoreactive (Figure 7E); 28.6% (2/7) reacted with gp140 (Figure 7F). These results indicate that the enriched VH replacement products in the plasmablast repertoires of chronically HIV-infected individuals may contribute to the generation of poly/autoreactive Abs.

## Discussion

In the present study, we demonstrated that plasmablast-derived Abs from chronically HIV-infected individuals with ART experience have weak anti-gp140-binding affinity and are non-neutralizing. Strikingly, these mAbs were predominantly polyreactive and autoreactive. We also identified NMDAR-DWEYS as an autoantigen target of the polyreactive/autoreactive mAbs, and such cross-reactivity induced apparent SH-SY5Y cell apoptosis *in vitro*. Moreover, the plasmablast IgH repertoires of chronically HIV-infected individuals differed from those of controls in that the frequencies of VH4-34 gene usage and VH replacement were higher.

Previous evidence suggested that although only 10–25% of HIV-1-infected individuals develop bnAbs (44), up to 50% of infected individuals can develop bnAb responses of low-to-moderate potency (45). Another study reported that 1% of infected patients exhibit unusually potent activity against a majority of clades, representing elite neutralizers (46). Our study is exceptional in that the study subjects are representative of the remaining patients that do not have the ability to generate bnAbs and control HIV/AIDS progression. In fact, all of the patients enrolled in this study experienced ART and maintained a stable, fairly low viral load and moderate CD4^+^ T-cell counts. We observed that the plasmablast-derived mAbs from chronically HIV-infected individuals were non-gp140- or weak gp140-binding Abs and non-neutralizing. Such findings are in agreement with previous studies demonstrating that individuals with untreated acute or advanced chronic HIV infection with higher viral loads were more likely to generate bnAbs (31, 47, 48). Although the sample size is limited, the results derived from such subjects are profound in that they may provide insights for practical and feasible vaccine designs.

The present study revealed the striking finding that randomly collected circulating plasmablasts from HIV-infected individuals predominantly produce poly/autoreactive Abs regardless of their gp140-binding reactivity. This underscores the importance of polyreactivity, which has been reported in gp120- or gp41-binding Abs (20, 49). However, the present study was designed differently from previous studies, which focused on HIV-specific B cells or Abs and HIV-infected individuals with a higher potential for generating bnAbs naturally. We collected the circulating plasmablasts randomly without pre-evaluation of HIV-antigen specificity. The results based on such cells reflect the characteristics of the whole pool of plasmablasts in chronically HIV-infected individuals unable to generate bnAbs through natural immunity. Therefore, we speculate that the predominant production of polyreactive/autoreactive Abs by circulating plasmablasts is another unrecognized dysfunction of B cells in chronically HIV-infected individuals.

For the polyreactive Abs obtained in this study, the dsDNA reactivity was strongly correlated with the reactivity against ssDNA, LPS, and insulin but only weakly correlated with their gp140 reactivity. Among the 35 gp140-binding Abs, there was a stronger correlation between polyreactivity and gp140-binding reactivity. Furthermore, the four Abs with relatively high gp140-binding capacities were all highly polyreactive. These findings support the assumption that anti-gp140 Abs may be selected from a pre-existing pool of polyreactive B cells before HIV infection. We therefore hypothesize that with continuous stimulation by HIV viral antigens, B cells expressing low-affinity polyreactive receptors that react with HIV antigens may be activated and positively selected to produce high-affinity anti-HIV Abs. It will be essential to determine how to induce and select such B cells with low-affinity polyreactive anti-HIV receptors. During normal B-cell development, B cells expressing high-affinity poly/autoreactive BCRs are removed from the repertoire at different early developmental checkpoints to establish B-cell tolerance (24). Defects in B-cell tolerance checkpoints may induce production of autoreactive Abs, leading to autoimmune diseases (50, 51). Poly/autoreactive Abs are frequently identified in autoimmune disease models (52). The predominant production of high-affinity polyreactive Abs by circulating plasmablasts in chronically HIV-infected individuals may reflect defects in B-cell tolerance checkpoints. It will be intriguing to further test this idea by characterizing the functional Ab repertoires of B cells at different developmental stages in HIV-infected individuals, especially in HIV-infected elite controllers. The results from such studies may provide new information regarding whether disrupting B-cell tolerance is necessary and sufficient to produce bnAbs.

Previous studies have shown that several bnAbs, including 2F5 and 4E10, are polyreactive against common autoantigens (19). These findings led to the hypothesis that B-cell tolerance programs prevent the generation of such neutralizing Abs (19, 53). Indeed, in mice carrying knocked-in IgH and IgL genes encoding the 2F5 or 4E10 Abs, B cells expressing 2F5 and 4E10 Abs were eliminated at multiple steps during early development (54, 55). Further analyses found that the 2F5 and 4E10 Abs recognize human kynureninase (KYNU) and splicing factor 3b subunit 3 (SF3b), respectively (56), which provides a partial explanation for the elimination of B cells expressing 2F5 or 4E10 receptors during development due to their cross-reactivity to cellular antigens. Recently, an in-depth analysis of 22 currently available bnAbs on high-density protein arrays further revealed that 45% are polyreactive against various protein antigens (57). In contrast, analysis of a large number of cloned recombinant mAbs from HIV gp140-binding memory B cells showed that nearly 75% of Env-specific mAbs are polyreactive (20). Interestingly, 70% of the recombinant Abs lost their gp140-binding capability but retained their polyreactivity after reversion of the mutated sequences to germline genes (20), suggesting that these anti-gp140 Abs are selected from a pool of low-affinity polyreactive Abs. Moreover, analysis of Abs obtained from acutely HIV-infected individuals also showed that a high proportion of gp41-reactive Abs are polyreactive (49). The comparatively lower VH mutation rate here was different from that of the gp140-specific Abs cloned from selected HIV-infected patients with broadly neutralizing activity (31), supporting the idea that Abs evolved to achieve broadly neutralizing potency might undergo persistent hypermutation and selection during chronic immune responses against HIV.

Cross-reaction of anti-dsDNA Abs with NMDAR-DWEYS is a recognized mechanism of NPSLE (38, 58). Determining whether the polyreactive/autoreactive Abs with minimal or no neutralizing abilities present in the circulation of HIV-infected patients may also be pathological is thus vitally important. With the introduction of highly active ART (HAART), the medical morbidity and mortality of individuals with HIV infection decreased dramatically; however, HIV-associated autoimmune symptoms remain burdensome (59). For instance, neuropsychological deficits persist in half of patients under HAART treatment (60). Interestingly, our data demonstrate that the poly/autoreactive Abs predominantly generated by the circulating plasmablasts of chronically HIV-infected individuals cross-react with NMDAR-DWEYS and induce evident SH-SY5Y cell apoptosis. Such findings indicate that NDMAR-cross-reactive ANAs may mediate neuronal damage *via* a similar pathway to that in NPSLE. Here, we enrolled asymptomatic patients who relied on ART to control HIV/AIDS pathogenesis, which limited our further investigations. We suspect that these newly identified NDMAR-cross-reactive ANAs, given sufficiently high titers and appropriate environments, would eventually damage the neuronal functions of the patients. Furthermore, we believe that it will be informative to investigate the CSF and serum titers of such Abs and conduct correlation studies with more patients presenting with the corresponding manifestations.

Currently, it is not clear why circulating plasmablasts in chronically HIV-infected individuals predominantly produce poly/autoreactive Abs, though some potential evidence is present in the repertoire analyses. We noted that longer HCDR3 is associated with stronger reactivity to dsDNA within the genes expressing Abs that have been tested for reactivity. We could not detect evidence of preferential gene usage in recombination events, usage of more charged aa, and higher VH mutation rate in the polyreactive Ab repertoire. It is possible that the small number of Ab gene sequences used in these analyses limited the identification of mechanisms that contribute to polyreactivity. Interestingly, our data demonstrated a bias favoring the utilization of the VH4-34 and VH1-2 genes in the IgH repertoires of the HIV-infected group. Notably, VH4-34-expressing cells are present at high proportions among SLE plasma cells, and skewed VH4-34 utilization is strongly associated with autoimmunity (61, 62) and cross-reactivity with commensal bacterial antigens (63). The VH1-2 gene has been reported to be associated with autoimmunity in lymphoproliferative neoplasms (64). Although limited data are available regarding the functionality of such VH genes and their encoding Abs, the predominance of VH4-34 and VH1-2 germline heavy chain usages is especially interesting in the context of the simultaneous predominance of polyreactive and autoreactive Abs in the mAb pool derived from the circulating plasmablasts of chronically HIV-infected individuals.

In addition to the biased VH gene usage, we saw other indicators of differences that might be associated with the predominance of polyreactivity/autoreactivity in the repertoire analyses. It has long been recognized that natural Abs responsible for combating various foreign pathogens retain poly/autoreactivity (52, 65). However, most of these natural Abs are encoded by germline IgH genes with low mutation rates, and these Abs display low affinity to various antigens (66, 67). In contrast, the polyreactive Abs that we cloned from circulating plasmablasts in HIV-infected individuals had relatively high affinities against different antigens, indicating that they had been positively selected during multiple rounds of germinal center reaction. Here, we demonstrated a significant enrichment of VH replacement products in the plasmablast repertoires of chronically HIV-infected individuals. Normally, VH replacement occurs during the pre-B-cell stage (68) and contributes to approximately 5% of the primary B-cell repertoire (69). VH replacement products are highly enriched in IgH genes derived from autoimmune-prone mice (70) and patients with autoimmune diseases or viral infections (71), as well as in IgH genes encoding different anti-HIV neutralizing Abs (72). The VH replacement products identified in the IgH genes in chronically HIV-infected individuals share the same prominent features as those previously reported: elongated IgH CDR3 regions and accumulated negatively charged aa encoded by the VH replacement footprints. Abs with such features tend to be autoreactive. This propensity is clear in the seven Abs encoded by VH replacement products from the plasmablasts of HIV-infected individuals; most of these Abs were polyreactive (4/7) and ANA-positive (6/7). Two of the seven VH replacement products encoded anti-gp140 Abs. Following this reasoning, VH replacement may be an effective machinery for generating desired IgH genes with targeted specificity.

In summary, the present study demonstrated that the circulating plasmablasts in chronically HIV-infected individuals predominantly produce poly/autoreactive Abs and that such polyreactivity/autoreactivity is not limited to HIV-specific Abs. These polyreactive/autoreactive Abs are also cross-reactive with NMDAR-DWEYS and detrimental to neuronal cell viability. Multiple factors, including longer HCDR3 regions, the accumulation of the repertoire shift, and VH replacement, may contribute to the evolution and survival of such cross-reactive Abs. Production of polyreactive/autoreactive Abs may represent another dysfunction of B cells during chronic HIV infection. Rational vaccine design eliciting polyreactive Abs to enhance the efficiency of producing bnAbs may therefore not be applicable to the entire HIV/AIDS population.

## Ethics Statement

All human samples were collected with written informed consent under protocols approved by the University of Nebraska Medical Center Institutional Review Board (IRB).

## Author Contributions

HL, CT, KS, and ZZ designed the research. HL, Yangsheng Yu, SL, and Yinshi Yue performed the research. ZZ, KS, HL, and Yangsheng Yu analyzed the data. HL, CT, KS, and ZZ wrote the manuscript. All authors revised the manuscript.

## Conflict of Interest Statement

The authors declare that the research was conducted in the absence of any commercial or financial relationships that could be construed as a potential conflict of interest.

## Supplementary Material

Figure S1 Representative of control (top) and HIV-infected individuals (bottom) showing the gating strategy used to sort single plasmablast. Digits in blue represent the percentage of plasmablast in CD19^+^ B cells.

Figure S2 **(A)** CD4^+^ T-cell counts and **(B)** percentages of plasmablast in CD19^+^ B cells in control donors and chronically HIV-infected individuals. Error bars indicate mean ± SEM. **(C,D)** Correlation of CD4^+^ T-cell count **(C)** and viral load **(D)** with percentage of plasmablast in CD19^+^ B cells of chronically HIV-infected individuals.

Figure S3 Monoclonal antibodies (mAbs) with DWEYS-cross-reactivity. **(A)** Histograms showing mAbs binding to SH-SY5Y cells measured by flow cytometry. **(B)** Viabilities of SH-SY5Y cells treated with mAbs with DWEYS-cross-reactivity at increasing concentrations. **(C)** Viabilities of SH-SY5Y cells treated with increasing molar ratio of DWEYS to each individual mAb. U78Abs was set as the negative control. Results were averaged from data of triplicate experiments. Error bars indicate mean with SD.
