# Supplementary figures and images for: Circulating Plasmablasts from Chronically Human Immunodeficiency Virus-Infected Individuals Predominantly Produce Polyreactive/Autoreactive Antibodies
Source: Front Immunol. 2017 Dec 6;8:1691. doi: 10.3389/fimmu.2017.01691 (PMC5723652; doi:10.3389/fimmu.2017.01691)

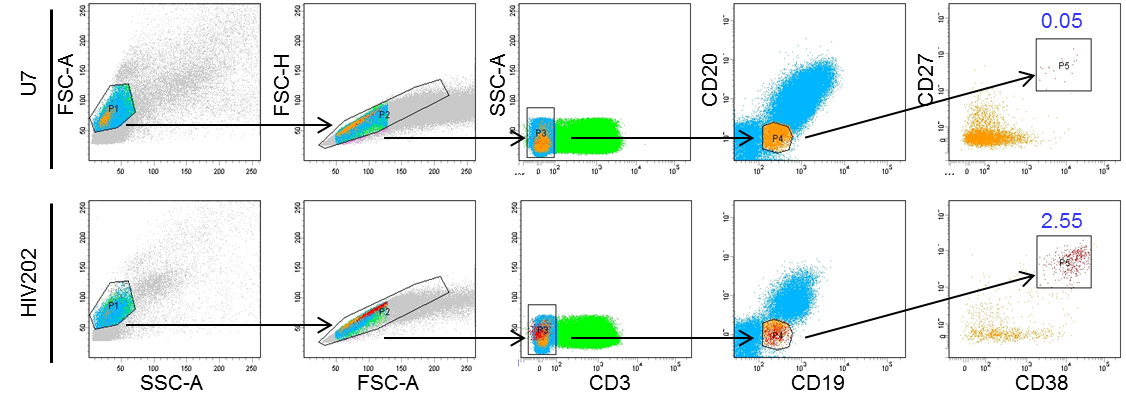

Supplement: Figure S1 — Representative of control (top) and HIV-infected individuals (bottom) showing the gating strategy used to sort single plasmablast. Digits in blue represent the percentage of plasmablast in CD19+ B cells. [file Image_1.tif]

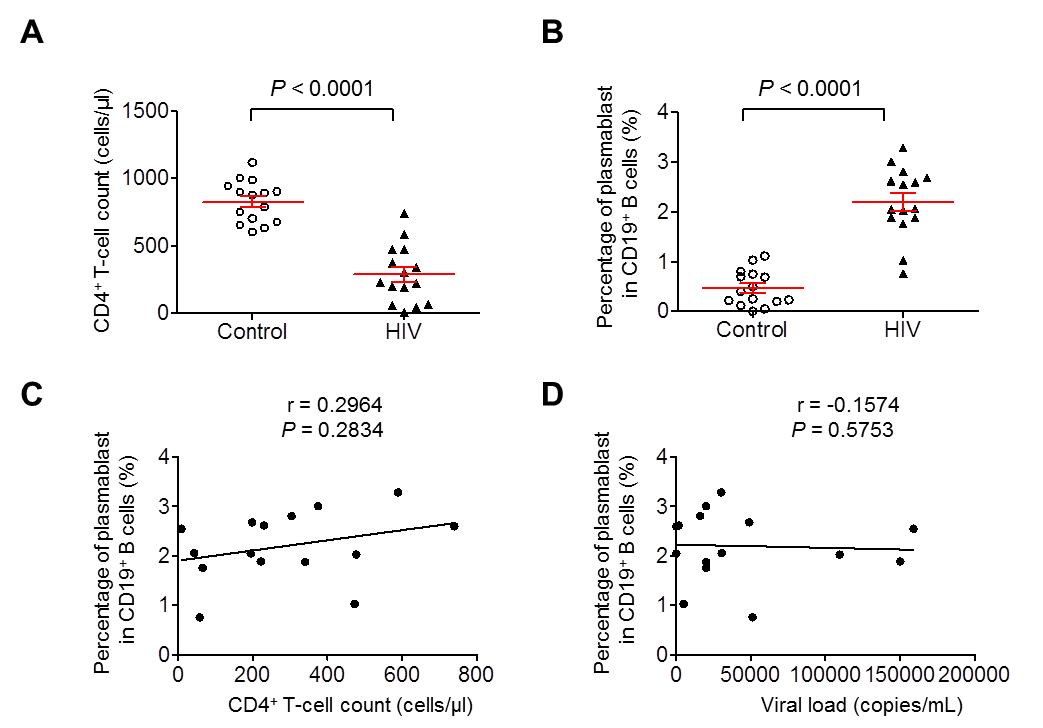

Supplement: Figure S2 — (A) CD4+ T-cell counts and (B) percentages of plasmablast in CD19+ B cells in control donors and chronically HIV-infected individuals. Error bars indicate mean ± SEM. (C,D) Correlation of CD4+ T-cell count (C) and viral load (D) with percentage of plasmablast in CD19+ B cells of chronically HIV-infected individuals. [file Image_2.tif]

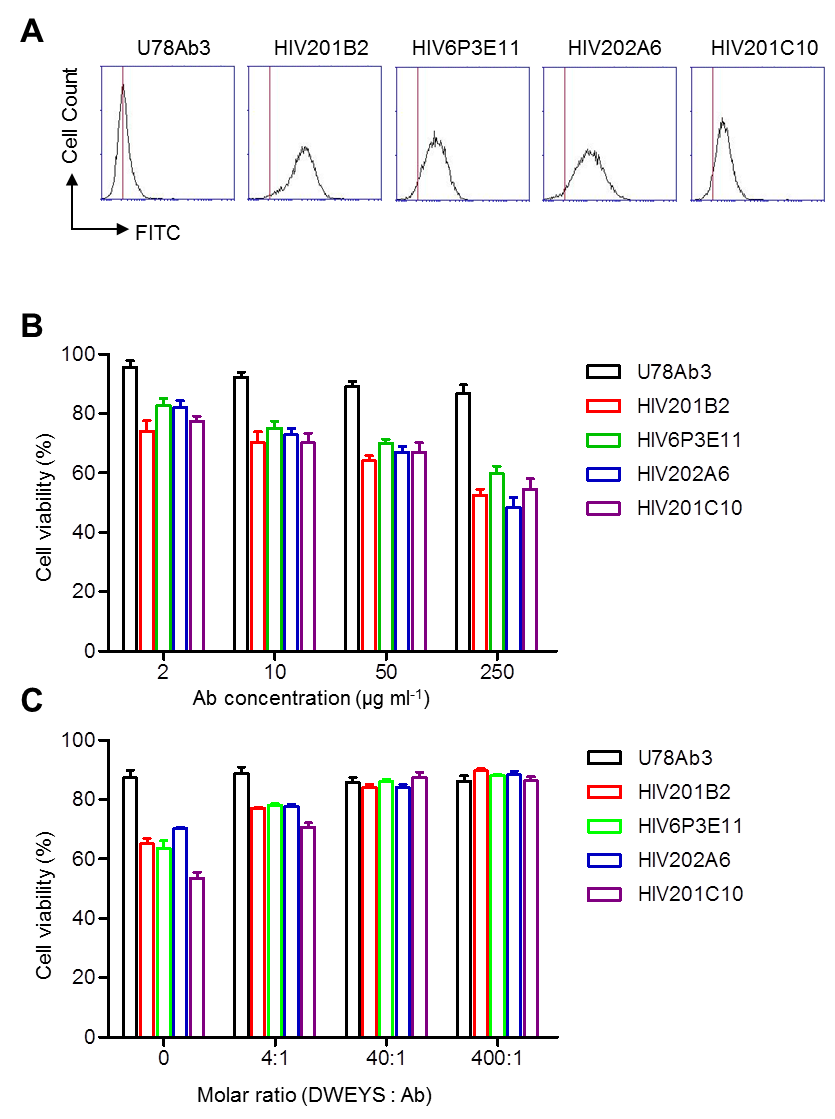

Supplement: Figure S3 — Monoclonal antibodies (mAbs) with DWEYS-cross-reactivity. (A) Histograms showing mAbs binding to SH-SY5Y cells measured by flow cytometry. (B) Viabilities of SH-SY5Y cells treated with mAbs with DWEYS-cross-reactivity at increasing concentrations. (C) Viabilities of SH-SY5Y cells treated with increasing molar ratio of DWEYS to each individual mAb. U78Abs was set as the negative control. Results were averaged from data of triplicate experiments. Error bars indicate mean with SD. [file Image_3.tif]
